# Protective effect of Flt3L on organ structure during advanced multiorgan dysfunction syndrome in mice

**DOI:** 10.3892/mmr.2015.3328

**Published:** 2015-02-10

**Authors:** GUANG TIAN, JIANGYANG LU, HUIQIN GUO, QIAN LIU, HONGWEI WANG

**Affiliations:** 1 Department of Pathology, The First Affiliated Hospital of General Hospital of PLA, Beijing 100048, P.R. China; 2 State Key Laboratory of Pathogen and Biosecurity, Beijing Institute of Microbiology and Epidemiology, Beijing 100071, P.R. China; 3 Department of Thoracic Surgery, Peking Union Medical College Hospital, Beijing 100730, P.R. China

**Keywords:** multiorgan dysfunction syndrome, fms-related tyrosine kinase 3 ligand, dendritic cells, pathology

## Abstract

The present study aimed to examine whether fms-related tyrosine kinase 3 ligand (Flt3L) protects the organs of mice with multiorgan dysfunction syndrome (MODS). Male C57BL/6 mice were randomly assigned to normal control, MODS and Flt3L treatment groups. The mouse models of MODS were established using intraperitoneal zymosan injections, followed by normal saline injections. The treatment group received 5 *μ*g/kg Flt3L for seven days, beginning on day five following zymosan injection. On day 12, the mortality rates of the Flt3L treatment and the MODS groups were 7 and 18%, respectively. Marked pathological changes were observed in the liver, lungs, kidneys and heart of the mice with MODS, including degeneration and focal necrosis of parenchyma cells. Mild pathological changes were observed in different organs of the Flt3L-treated mice. In the MODS group, the number of CD4^+^ T lymphocytes was significantly reduced, whereas the number of CD8^+^ T lymphocytes was significantly increased compared with that in the normal control group; thus, the CD4^+^/CD8^+^ ratio was reduced. In the Flt3L treatment group, the average number of CD4^+^ T lymphocytes was not significantly different to the average number of CD4^+^ T lymphocytes in the normal group. In conclusion, Flt3L administration improved the immune status and alleviated the organ damage in mice with late-phase MODS.

## Introduction

Multiorgan dysfunction syndrome (MODS) commonly occurs subsequent to severe burns, trauma or major surgical stress. It is currently a leading cause of mortality in critically ill patients and has become a complicated problem in surgery (1,2). MODS refers to the dysfunction or failure of two or more systems or organs and occurs 24 h after the body suffers severe trauma, shock, infection or acute injury, which results in multiple organs being unable to maintain a stable internal environment (3). In 1991, the joint conference committee of the American College of Chest Physicians and the Society of Critical Care Medicine established the concept of systemic inflammatory response syndrome and the corresponding compensatory anti-inflammatory response (4). It is thought that a series of chain reactions or cascade effects arising from an imbalance between pro-inflammatory and anti-inflammatory mediators leads to the development of MODS; thus, the severity and fate of MODS depend on the balance between the two mediators (5). Currently, neither effective treatments nor preventive measures against MODS are available. Studies regarding the pathogenesis of MODS have focused on immunological function disorder, which is important in the occurrence and development of MODS (6). During severe injury and infection, large quantities of inflammatory mediators are released, resulting in tissue deterioration and exudation, whereas continuously increasing levels of mediators, such as cytokines, place the immune system in a bipolar state of activation and paralysis. Therefore, the high expression levels of immunological mediators jointly result in tissue injury and the occurrence and development of MODS. Immunological studies have demonstrated that the main manifestations of 'immunological paralysis' in MODS are caused by the human leukocyte antigen DR, which continuously reduces intracellular defects, including the proliferation of antigen-specific T lymphocytes. T lymphocytes have been shown to be inhibited by the reduced expression of major histocompatibility complex II (I-A^b^, also termed MHC-II) antigen (6–8). These defects include the loss of important cell surface antigen expression, cytokine dysregulation, changes in antigen expression levels and acceleration of cytokine apoptosis (9–14).

A previous study revealed that in advanced MODS, the reduction in dendritic cell activity and the resulting massive lymphocyte apoptosis are important in immunosuppression and MODS formation (15). Relieving the immune imbalance and immunosuppression at this stage may prevent the occurrence of MODS.

Fms-related tyrosine kinase 3 ligand (Flt3L) is a cytokine that promotes the cytopoiesis and differentiation of various stem cells, blood cells and precursor blood cells, and it promotes the proliferation, differentiation and maturation of prolymphocytes, dendritic cells, natural killer cells and B lymphocytes (16–18). One study found that Flt3L reversed endotoxin-induced septic immunological paralysis in mice (19). In the present study, the zymosan-induced MODS model was used, which is currently employed to simulate MODS in clinical patients. Flt3L was injected into mice with advanced MODS to evaluate whether it exhibited a protective effect on the mice.

## Materials and methods

### Model preparation

Specific pathogen-free-grade male C57BL/6 mice, aged six to eight weeks with body weights ranging from 20–25 g, were purchased from the Experimental Animal Center of the Academy of Military Medical Sciences (Beijing, China). As determined by a previously published procedure (3), 1 g zymosan powder (Sigma-Aldrich, St. Louis, MO, USA) and 40 ml medicinal liquid paraffin were mixed using a high frequency magnetic stirrer for 1 h to prepare a 25 g/l zymosan suspension. Subsequently, the suspension was sterilized in 100°C water for 80 min and then cooled to room temperature. The abdominal skin areas of the mice were locally sterilized and 1 mg/g zymosan suspension was administered intraperitoneally. The present study was conducted in strict accordance with the recommendations of the Guide for the Care and Use of Laboratory Animals of the National Institutes of Health. The animal use protocol has been reviewed and approved by the Institutional Animal Care and Use Committee of the First Affiliated Hospital of General Hospital of PLA (Beijing, China).

### Animal treatment

The experimental animals were randomly divided into a normal control group (10 mice), an MODS group (30 mice) and an Flt3L treatment group (30 mice). In the MODS group, normal saline was intraperitoneally injected once a day for seven days beginning from day five after the zymosan injection. For the Flt3L treatment group, recombinant mouse Flt3L (5 *μ*g/kg; CytoLab Ltd., Rehovot, Israel) was intraperitoneally injected once a day for seven days beginning on day five after zymosan injection. For the normal control group, normal saline was intraperitoneally injected once a day for seven days beginning on day five without zymosan intraperitoneal injection. Ten mice from each group were sacrificed and samples were collected on day 12 after zymosan injection.

### Detection of splenic dendritic cells (DCs)

The mouse spleen was held in a dry dish with forceps and 1 ml collagenase D solution (500 *μ*l; Sigma-Aldrich) was slowly injected by a 22-G needle. The spleen was then torn open with the needle and transferred to another dish. Subsequently, the spleen tissues were excised and incubated for 25 min at 37°C. A volume of 10 mM EDTA was added and the tissues were incubated continuously for 5 min. The tissues were ground on a 400-mesh metal screen and the cell liquid was collected and centrifuged for 10 min at 111 x g. A total of 2 ml red cell lysate (BD Biosciences, Franklin Lakes, NJ, USA) was added and the sample was inverted four times, incubated for 8 min in the dark and centrifuged for 4 min at 111 x g to remove the supernatant liquid. Subsequently, the cells were suspended in 2 ml phosphate-buffered saline (PBS). Following Trypan blue staining, the cells were smeared onto a blood cell counting board and the number of living cells was determined under light microscopy (ideally >95%). The cell concentration was adjusted to 1×10^6^ cells/ml. A total of 0.5 *μ*l mouse monoclonal anti-phycoerythrin (PE)-CD11c (N418; Biolegend, Inc., San Diego, CA, USA) and 0.5 *μ*l mouse monoclonal anti-I-A^b^-fluorescein isothiocyanate (FITC) (AF6-120.1; Biolegend, Inc.) antibodies were added to the cell mixture, which was mixed uniformly, allowed to stand for 30 min at 4°C, washed twice with 0.1 M PBS and fixed with 1% paraformaldehyde. Flow cytometry was used for detection.

### Phenotypic analysis of peripheral blood T lymphocytes

The animal was placed in a restraining device thus only the tail was exposed for drawing blood. The tail was warmed by a heat lamp in order to dilate the vessels, sterilized with 70% ethanol and 1–2 ml blood was drawn from the caudal vein with a 23G needle, prior to sacrifice of the animals.

Mouse anticoagulant (100 *μ*l, 10.0–12.5 IU/ml) was placed into three test tubes containing anti-CD3-FITC, anti-CD4-phycoerythrin (PE), anti-CD8-PE-Cy5 and anti-I-A^b^-FITC, respectively (0.5 *μ*l/10^6^ cells; all from Becton Dickinson Biosciences, San Jose, CA, USA), mixed uniformly and allowed to stand for 30 min at room temperature. Whole blood cells were reacted with antibody and the blood cells were then lysed using a double volume of red cell lysate, following which the solution was immediately mixed evenly. After 15 min at room temperature, the mixture was centrifuged for 4 min at 187 x g to remove the supernatant liquid. The cells were resuspended in 1% paraformaldehyde. All data were acquired using FACScalibur (BD Biosciences) and analyzed using CellQuest Pro software version 4.0 (BD Biosciences).

### I-A^b^ labeling detection

Mouse anticoagulant (100 *μ*l, 10.0–12.5 IU/ml) was placed into a test tube containing 1 *μ*l anti-FITC-I-A^b^ antibody, mixed uniformly and left to stand at room temperature for 30 min. Subsequently, a double volume of red cell lysate was added and the solution was immediately mixed evenly. After 15 min at room temperature, the mixture was centrifuged for 4 min at 200 x g to remove the supernatant liquid. PBS was added, and flow cytometry was used to observe the quality and percentage of I-A^b^-labeled mononuclear cells.

### Pathological examination under light microscopy

The liver, lung, kidney and heart tissues of mice from the different groups were collected and fixed with 10% neutral formaldehyde solution for 24 h. The samples were dehydrated by a tissue-dehydrating machine, embedded in paraffin and sectioned into 4-*μ*m thick slices. Following roasting, slicing and dewaxing, routine hematoxylin-eosin staining was conducted and the sections were observed under light microscopy using an Olympus BX40F microscope (Olympus, Melville, NY, USA). Images were captured with a Sony 3CCD color video camera (Sony, Tokyo, Japan).

### Transmission electron microscopy

Fresh spleen tissues from each of the groups were sectioned into 1-mm tissue blocks. The tissue blocks were immediately placed in 3% glutaric dialdehyde, premixed for 24 h, fixed with OsO_4_ for 2 h, dehydrated with a gradient ethanol and acetone, embedded in Epson 812 and sectioned with an LKB-V ultramicrotome. Following double staining with uranyl acetate and lead citrate, the sections were viewed under a JEM-1200EX transmission electron microscope (JEOL, Tokyo, Japan).

### Statistical analysis

SPSS statistical analysis software (version 10; SPSS, Inc., Chicago, IL, USA) was used for data processing and data are expressed as the mean ± standard deviation. Student’s t-test was used for statistical analysis of the data. P<0.05 was considered to indicate a statistically significant difference.

## Results

### Comparison of animal mortality rate

At day 12 following intraperitoneal zymosan injection, the mice with MODS presented with somnolence, dyspnea, depression, eye closing, dirty fur, chills and anorexia. The mortality rate in this group was 18%, whereas the mortality rate in mice in the Flt3L treatment group was only 7%.

### Pathological changes in main organs

**Figure 1:**
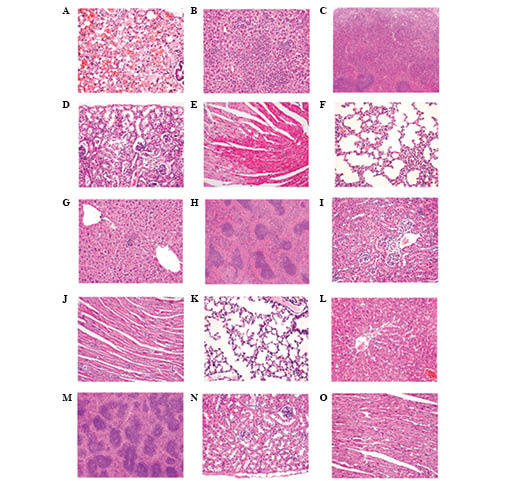
Pathological changes in the main organs. Mice in the multiorgan dysfunction syndrome (MODS; n=30) group presented: (A) Severe pulmonary edema complicated with hemorrhoea; (B) focal necrosis of liver cells; (C) disappearence of splenic white pulp and reduced acini; (D) renal tubular epithelial vacuolar degeneration and glomerular atrophy; (E) partial myocardial fibers greatly dyed red, shrunken and degenerated. Mice in the Flt3L group (n=30) presented: (F) Alveolar septum broadening and inflammatory cell infiltration; (G) occasional point necrosis of liver cells; (H) plentiful splenic white pulp lymphocytes and acini restored to normal level; (I) almost normal appearance of the kidney; (J) almost normal appearance of the heart. (K–O) Tissue sections in the normal control group (n=10) did not exhibit any histopathological lesions: (K) Lung; (L) liver; (M) spleen; (N) kidney; (O) heart. The lung, liver, kidney, spleen and heart tissues were stained with hematoxylin-eosin and observed under light microscopy (magnification, ×200).

The liver, lungs, kidneys, heart and spleen of the mice with MODS exhibited marked pathological changes, whereas the pathological changes in the Flt3L treatment mice appeared less severe (Fig. 1).

The lungs of mice with MODS exhibited alveolar edema complicated with hemorrhage as well as pulmonary interstitial edema complicated with focal inflammatory cell infiltration. Alveolar septum thickening and interstitial pulmonary perivascular lymphocytic infiltration were also observed (Fig. 1A). In the Flt3L treatment group, the lesions were markedly less pronounced; pulmonary interstitial infiltration and inflammatory cell infiltration of the alveolar septum were occasionally visible (Fig. 1F).

The liver cells of mice with MODS presented with albuminoid degeneration and vacuolar degeneration. Locally, eosinophilic variants and spotty liver necrosis were observed; proliferating hepatic sinus Kupffer cells and further inflammatory cell infiltrations were also identified (Fig. 1B). In the Flt3L treatment group, the liver only presented with focal eosinophilic variants of liver cells (Fig. 1G).

In the kidneys, the glomeruli in the MODS group exhibited hyperemic and ischemic changes, and the renal tubular epithelial cells exhibited albuminoid degeneration, granular degeneration and partial epithelial vacuolar degeneration. Local interstitial perivascular edema and lymphocyte infiltrations were also observed (Fig. 1D). In the Flt3L treatment group, only partial glomerular hyperemia and local renal tubular epithelial albuminoid degeneration were visible (Fig. 1I).

In the heart, the MODS group presented with myocardial interstitial edema with vasodilatation and hyperemia. Partial myocardial fibers shrank and appeared red, with a condensed sarcoplasm, increased acidophilia and a flaky distribution (Fig. 1E). In the Flt3L treatment group, these lesions were clearly less severe and only a few myocardial fibers exhibited focal degeneration (Fig. 1J).

In the spleen, the white pulp in the MODS group appeared shrunken, the periarterial lymphatic sheath had disappeared and lymphocytic maturation was greatly reduced. In addition, acini were rare, the follicular germinal centers were not clearly apparent, and the boundary between the white pulp and the red pulp was unclear. In the red pulp, neutrophilic infiltration was observed (Fig. 1C). In the Flt3L treatment group, the number of white pulp lymphocytes was clearly increased and the number of acini was close to that of the normal group (Fig. 1H). The tissues of the normal groups, including the lung, liver, spleen, kidney and heart, exhibited no histopathological lesions (Fig. 1K–O).

### Phenotype analysis of peripheral blood T lymphocytes

**Figure 2:**
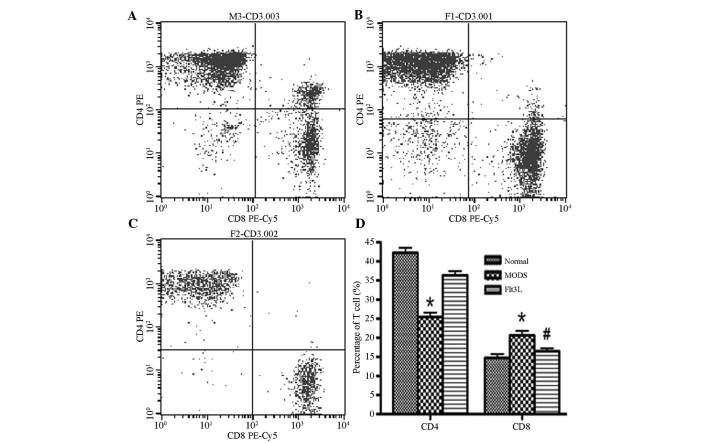
Changes of peripheral blood T lymphocyte subpopulation in peripheral blood. (A) Normal group; (B) MODS group; (C) Flt3L treatment group. (D) Cumulative results from different groups. ^*^P<0.05 compared with the normal control group; ^#^P<0.05 compared with the MODS group. MODS, multiorgan dysfunction syndrome; Flt3L, fms-related tyrosine 3 ligand.

In the MODS group, the number of CD4^+^ T cells was significantly reduced, whereas the number of CD8^+^ T cells was significantly increased in comparison with the normal control group (P<0.05). In the Flt3L treatment group, the number of CD4^+^ T cells was increased in comparison with the MODS group and approached normal levels, whereas the number of CD8^+^ T cells was significantly reduced in comparison with the MODS group (P<0.05; Fig. 2).

### I-A^b^ labeling

**Figure 3:**
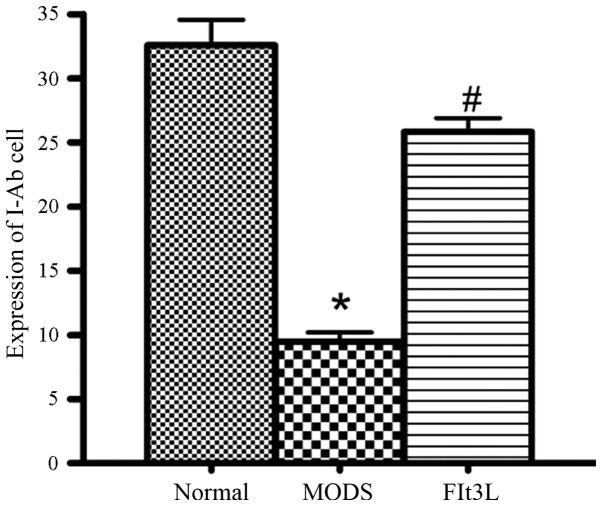
I-A^b^ expression in peripheral blood mononuclear cells. Peripheral blood was collected from normal control mice, mice with MODS and Flt3L treated mice. For I-A^b^ expression, blood cells were labeled with I-A^b^ antibodies. The results are presented as the percentage of cells positively labeled with antibodies. ^*^P<0.01 compared with the normal control group; ^#^P<0.01 compared with the MODS group. MODS, multiorgan dysfunction syndrome; Flt3L, fms-related tyrosine 3 ligand; I-A^b^, major histocompatibility complex-II.

In the MODS group, the I-A^b^ expression levels in the peripheral blood were significantly lower than those in the normal control group (P<0.01). In the Flt3L treatment group, the I-A^b^ expression levels in the peripheral blood were significantly higher than those in the MODS group (P<0.01) and close to normal levels. This result indicated that the zymosan-induced reduction in the immune activity of antigen presenting cells and DCs was restored following Flt3L treatment (Fig. 3).

### Immunophenotype analysis of splenic DCs

**Figure 4:**
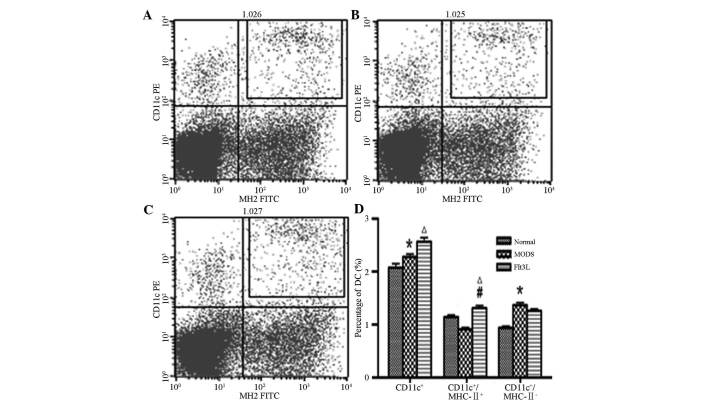
Expression percentages of CD11c and I-A^b^ on the mouse spleen DC surface. Splenocytes were isolated and stained to assessed the expression levels of CD11c/I-A^b^. Representative flow cytograms of splenocytes are presented for (A) the normal group, (B) MODS group and (C) Flt3L treatment group. (D) Cumulative results from different groups: Percentage of total DCs (% of CD11c^+^), percentage of mature DCs (% of CD11c^+^/I-A^b+^) and percentage of immature DCs (% of CD11c^+^/I-A^b−^). ^*,Δ^P<0.05 compared with the normal control group; ^#^P<0.05 compared with the MODS group. MODS, multiorgan dysfunction syndrome; Flt3L, fms-related tyrosine 3 ligand; MHC, major histocompatibility complex; DC, dendritic cell.

Compared with the normal control group, the total number of CD11c^+^ DCs and the number of immature DCs (CD11c^+^/I-A^b−^) in the MODS group were significantly increased (P<0.05). In the Flt3L treatment group, the total number of CD11c^+^ DCs along with the number of mature DCs (CD11c^+^/I-A^b+^) were significantly increased compared with those in the normal control group (P<0.05). The number of mature DCs (CD11c^+^/I-A^b+^) in the Flt3L treatment group was also significantly increased when compared with that in the MODS group (P<0.05). No significant differences were identified (P>0.05) between the numbers of immature DCs (CD11c^+^/I-A^b−^) in the Flt3L group and those in the MODS or the normal control group (Fig. 4).

### Ultrastructural observation of splenic DCs

**Figure 5:**
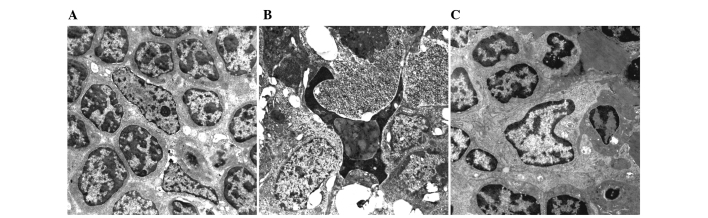
Ultrastructure of spleen. The spleen was collected from the MODS mice, the flt3L treatment mice and the control mice. The tissue were fixed in 3% glutaraldehyde solution and then post-fixed in 1% osmium tetroxide. Afterwards, the tissue samples were dehydrated with a serial alcohol gradient and then embedded in Epon 812. Ultrathin sections were stained with 1% uranyl acetate and lead citrate and examined under a JEM-1200EX transmission electron microscope. (A) Spleen DC of the normal group; (B) Spleen DC of the MODS group; (C) Spleen DC of flt3L group (EMx12,000). Arrow indicates dendritic cells.

In the normal control group, large volumes of irregularly or spindle shaped splenic DCs were observed. Other observations included the formation of several projections on the cell surface (extending towards gaps in the surrounding lymphocytes), reduced cytoplasmic electron density, underdeveloped organelles and lymphocytes centered on DCs (Fig. 5A). On day 12, the majority of splenic DCs in the MODS group presented with regressive and apoptotic changes, and the surrounding lymphocytes exhibited cytolytic and apoptotic degeneration (Fig. 5B). In the Flt3L treatment group, the splenic DCs were activated, with increased cell bodies, lengthened projections, increased organelles and lymphocytes centered on DCs (Fig. 5C).

## Discussion

Researchers have conducted a number of studies and developed clinical applications to prevent the progression of advanced MODS, however with unsatisfactory results. These strategies failed as they did not specifically target the underlying mechanism (1).

Flt3L is a cytokine that promotes the cytopoiesis and differentiation of various stem cells, blood cells and blood precursor cells. Flt3L synergistically functions with other cytokines and is a good amplification agent, although Flt3L does not influence cell morphology or the cell phenotype. *In vitro* studies have demonstrated that Flt3L promoted the differentiation of CD34^+^ hemopoietic stem cells from bone marrow and umbilical blood into DCs, and enhanced DC amplification resulting from GM-CSF and TNF-α induction, thereby increasing the number of DCs (15). *In vivo* studies have revealed that Flt3L increased the number of DCs in the spleen, bone marrow, lymph nodes and liver of mice (8,11–17).

The spleen is the largest peripheral immune organ and is an important site for lymphocytic activation and immune responses. The functions of peripheral immune organs are crucial in the maintenance of the immune response and inflammatory reaction balance.

I-A^b^ is a histocompatibility type-II antigen in mice, whose receptors are only present on the surface of activated antigen-presenting cells. Therefore, I-A^b^ expression directly reflects the activation of mouse antigen-presenting cells. CD11c, a sensitive DC marker, is expressed in mature and immature DCs. In the present study, the combined analysis of CD11c and I-A^b^ markers effectively differentiated mature DCs from immature DCs.

The surfaces of mononuclear cells in the peripheral blood of mice with advanced MODS exhibited significantly reduced I-A^b^ expression levels (P<0.01) and a considerably reduced CD4^+^/CD8^+^ T lymphocytic ratio compared with those in the normal control group, which indicated a severely reduced immune response. In the present study, splenic DCs still proliferated, but were predominantly immature DCs (CD11c^+^/I-A^b−^). The splenic DCs were shrunken and regressed with apoptotic and cytolytic changes. In addition, white pulp lymphocytes were absent. For the mice in the Flt3L treatment group, the splenic DC volume and the number of DC cells, which were predominantly mature DCs (CD11c^+^/I-A^b+^), were increased in comparison with those in the normal control group. The I-A^b^ expression levels in the peripheral blood mononuclear cells of the Flt3L treatment group were significantly higher than those in cells of the MODS group (P<0.01). In addition, the number of white pulp lymphocytes was evidently increased, along with the number of CD4^+^ T cells in the peripheral blood. The CD4^+^/CD8^+^ ratio was normal. Furthermore, the mortality rate of experimental mice was reduced from 18% in the MODS group to 7% in the treatment group, which suggests that the immunological functions in the mice were restored and improved following Flt3L treatment.

In the early stages of severe infections and MODS, immune organs and DCs in the peripheral blood exhibit high proliferation and activation, causing the body to produce an excessive immune response, thereby initiating MODS. Following a disease remission period of five to seven days, the number of DCs in the MODS stage remained increased, but with a markedly reduced immunological competence. Previous studies (3–5) considered that immunosuppression during MODS is possibly associated with massive consumption and apoptosis of DCs and lymphocytes during the early stages of the disease. Other studies (20–24) demonstrated that DCs are dual-directional immune regulators, inducing the immune response but negatively regulating the response to maintain homeostasis. DCs are divided into static, active and tolerant DCs as determined by function. The negative regulatory function of DCs is mainly performed by tolerant DCs. These induce an immune tolerance under physiological conditions, and immunosuppression and immunological paralysis under pathological conditions (25). Tolerant DCs perform negative regulation by inducing T-cell deactivation, immune response deflection, regulatory T-cell formation, and promotion of apoptosis of activated T cells and other routes (15). DCs between the remission stage and the end stage of MODS (12 days) are inactive or tolerant, and exhibit a negative regulatory effect, which results in the deactivation or suppression of T cells. In advanced MODS, DCs are deactivated and the direction of immune regulation changes.

In the remission stage of MODS, injected Flt3L reversed the activity of ‘tolerant DCs’ by amplifying the number of DC precursor cells and activating DC activity. DCs have been demonstrated to greatly enhance the cytotoxic efficacy towards tumor cells (26). Flt3L enhanced the ability of DCs to stimulate T cell activation to restore and strengthen cellular immunological function. In conclusion, the the present study suggested that Flt3L injections restore the immunological functions disrupted by MODS, thereby preventing the progression of MODS. Therefore, Flt3L may be suitable for further studies and clinical applications of immune prevention in MODS and sepsis.
